# Differential effects of intragastric acid and capsaicin on gastric emptying and afferent input to the rat spinal cord and brainstem

**DOI:** 10.1186/1471-2202-6-60

**Published:** 2005-09-14

**Authors:** Peter Holzer, Evelin Painsipp, Rufina Schuligoi

**Affiliations:** 1 Department of Experimental and Clinical Pharmacology, Medical University of Graz, Universitätsplatz 4, A-8010 Graz, Austria

## Abstract

**Background:**

Hydrochloric acid (HCl) is a potential threat to the integrity of the gastric mucosa and is known to contribute to upper abdominal pain. We have previously found that gastric mucosal challenge with excess HCl is signalled to the rat brainstem, but not spinal cord, as visualized by expression of c-fos messenger ribonucleic acid (mRNA), a surrogate marker of neuronal excitation. This study examined whether gastric mucosal exposure to capsaicin, a stimulant of nociceptive afferents that does not damage the gastric mucosa, is signalled to both brainstem and spinal cord and whether differences in the afferent signalling of gastric HCl and capsaicin challenge are related to different effects on gastric emptying.

**Results:**

Rats were treated intragastrically with vehicle, HCl or capsaicin, activation of neurons in the brainstem and spinal cord was visualized by in situ hybridization autoradiography for c-fos mRNA, and gastric emptying deduced from the retention of intragastrically administered fluid. Relative to vehicle, HCl (0.5 M) and capsaicin (3.2 mM) increased c-fos transcription in the nucleus tractus solitarii by factors of 7.0 and 2.1, respectively. Capsaicin also caused a 5.2-fold rise of c-fos mRNA expression in lamina I of the caudal thoracic spinal cord, although the number of c-fos mRNA-positive cells in this lamina was very small. Thus, on average only 0.13 and 0.68 c-fos mRNA-positive cells were counted in 0.01 mm sections of the unilateral lamina I following intragastric administration of vehicle and capsaicin, respectively. In contrast, intragastric HCl failed to induce c-fos mRNA in the spinal cord. Measurement of gastric fluid retention revealed that HCl suppressed gastric emptying while capsaicin did not.

**Conclusion:**

The findings of this study show that gastric mucosal exposure to HCl and capsaicin is differentially transmitted to the brainstem and spinal cord. Since only HCl blocks gastric emptying, it is hypothesized that the two stimuli are transduced by different afferent pathways. We infer that HCl is exclusively signalled by gastric vagal afferents whereas capsaicin is processed both by gastric vagal and intestinal spinal afferents.

## Background

Gastric acid-related diseases are among the most prevalent mucosal disorders of the upper gastrointestinal tract. There is also evidence that hydrochloric acid (HCl) contributes to the pain associated with gastro-oesophageal reflux and peptic ulcer disease as well as to non-cardiac chest pain and functional dyspepsia [1-3]. Gastric chemonociception evoked by exposure of the rat stomach to excess HCl is mediated by vagal afferent neurons, given that the visceromotor pain response to intragastric (IG) administration of HCl is abolished by bilateral vagotomy, whereas the visceromotor response to distension remains unaltered [4]. This finding is consistent with our observation that IG administration of HCl is signalled to the nucleus tractus solitarii (NTS) of the rat brainstem, the central termination area of vagal afferents, as visualized by expression of messenger ribonucleic acid (mRNA) for the immediate early gene c-fos, whereas no induction of c-fos mRNA is seen in the spinal cord [5,6]. Similarly, gastric challenge with ammonium hydroxide induces c-fos mRNA and protein only in the brainstem, but not spinal cord, of the rat [7]. While both HCl and ammonium hydroxide injure the gastric mucosa at concentrations that cause near-maximal translation of the c-fos gene in the NTS [7], capsaicin is a chemical that excites gastrointestinal afferent neurons [8-10] without causing damage to the rat stomach [11]. This is because capsaicin stimulates afferent neurons by gating transient receptor potential ion channels of vanilloid type 1 (TRPV1), which are expressed by both vagal and spinal afferent neurons innervating the rat stomach and intestine [12-16].

The overall aim of this exploratory study was to test whether gastric mucosal challenge with capsaicin and excess HCl is differentially transmitted to the rat brainstem and spinal cord and whether the differential processing of the two stimuli takes place at the level of the upper gastrointestinal tract. Two sets of experiments were performed to address these questions. In the first study, IG administered capsaicin and HCl were compared in their effects on the expression of c-fos mRNA in the NTS and in the caudal thoracic spinal cord which receives the densest afferent input from the rat stomach [17,18]. It was in particular investigated whether IG administration of HCl and capsaicin has a distinct effect on neurons in specific laminae and nuclei of the dorsal spinal cord. The concentrations of HCl (0.5 M) and capsaicin (0.64 and 3.2 M) tested in these experiments were selected from previous experiments. Exposure of the rat gastric mucosa to HCl (0.5 M) causes a distinctive but submaximal induction of c-fos mRNA in the brainstem [5], while IG administration of 0.64 mM capsaicin is maximally effective in increasing gastric mucosal blood flow in a sensory neuron-dependent manner [19].

Since it was found that the afferent signalling of HCl and capsaicin to the NTS and spinal cord is different, the aim of the second study was to examine whether HCl and capsaicin influence gastric motility and emptying in a differential manner. It was reasoned that the magnitude of the c-fos response in the NTS and spinal cord depends both on the concentration of the chemicals and the duration of their presence in the stomach. It has previously been found that IG administration of excess HCl inhibits gastric emptying and alters intragastric pressure [5,20]. The findings of this study reveal that, unlike excess HCl, capsaicin does not inhibit gastric emptying. It is hypothesized, therefore, that gastric HCl challenge is exclusively signalled to the brainstem via vagal afferents, because it is retained in the stomach for a prolonged period of time, whereas both gastric vagal and duodenal spinal afferents respond to IG administration of capsaicin, the emptying of which into the duodenum is not retarded.

## Results

### Effects of HCl and capsaicin to induce c-fos mRNA in the NTS and spinal cord (study 1)

**Figure 1:**
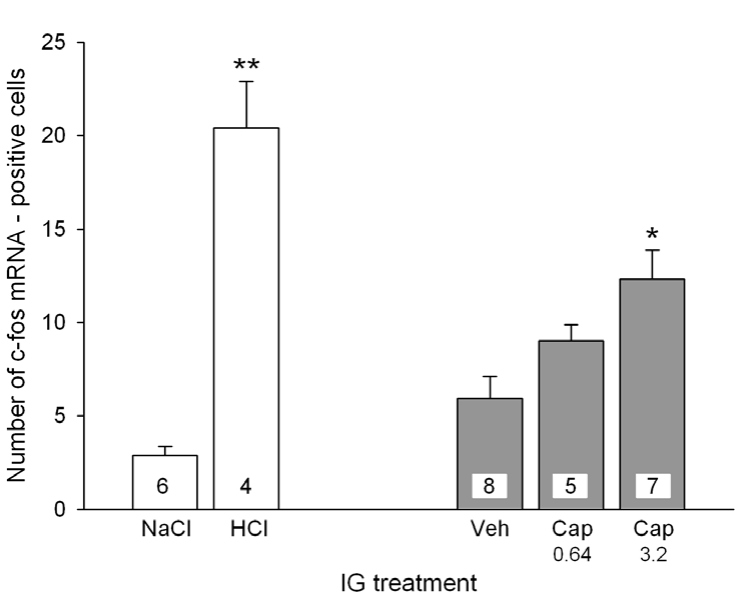
Number of c-fos mRNA-positive cells per section (0.01 mm) in the unilateral NTS determined 45 min after IG administration of NaCl (0.15 M), HCl (0.5 M), vehicle (Veh) and capsaicin (Cap, 0.64 and 3.2 mM). Means + SEM, n as indicated. * P < 0.05 versus Veh, ** P < 0.01 versus NaCl.

As illustrated previously [5,21], IG administration of 0.5 M HCl caused many neurons in the NTS to express c-fos mRNA when compared with IG administration of physiological saline. The number of c-fos mRNA-positive cells per section seen after IG administration of HCl was 7.0 times larger than after IG administration of physiological saline (Figure 1). The number of c-fos mRNA-positive neurons per NTS section counted after IG administration of vehicle tended to be higher than after administration of physiological saline, although this effect was statistically not significant (Figure 1). Relative to vehicle, capsaicin (0.64 and 3.2 mM) enhanced the number of c-fos mRNA-positive neurons per NTS section, this effect depending on the concentration of the drug. As can be seen in Figure 1, only the concentration of 3.2 mM capsaicin was able to significantly increase the induction of c-fos mRNA by a factor of 2.1. The distribution of c-fos mRNA-positive cells in the NTS after IG exposure to HCl (0.5 M) and capsaicin (3.2 mM) was uneven, the highest number of activated cells occurring in the ventromedial part of the NTS [22].

**Figure 2:**
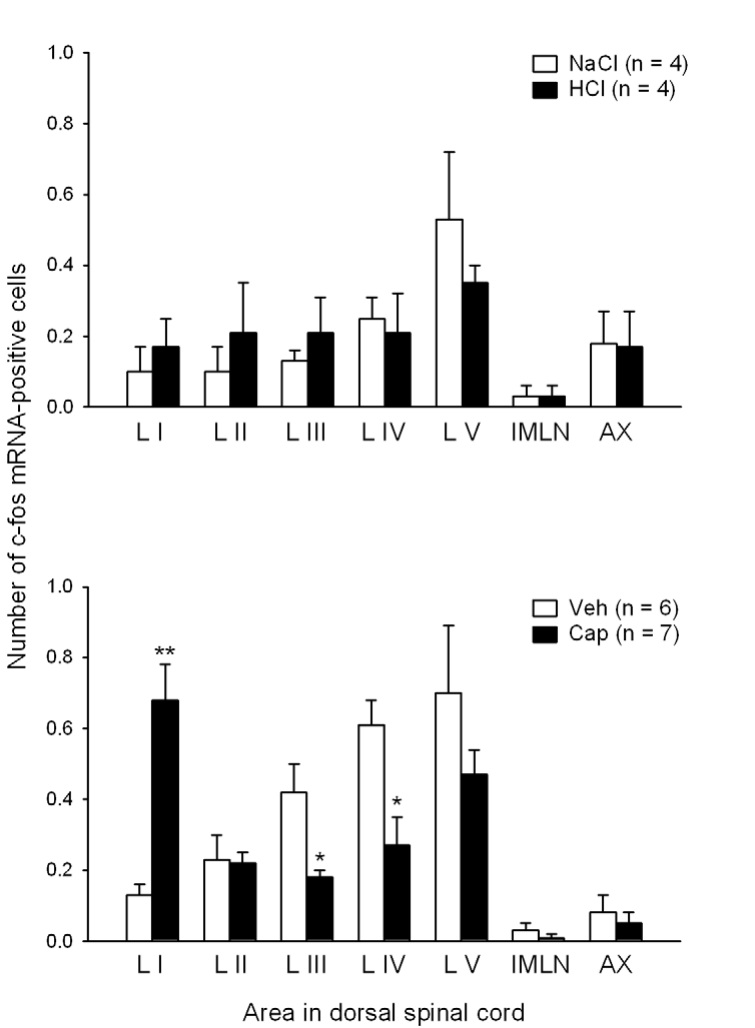
Number of c-fos mRNA-positive cells per section (0.01 mm) in various laminae and areas of the unilateral dorsal half of the caudal thoracic spinal cord determined 45 min after IG administration of (A) NaCl (0.15 M), HCl (0.5 M), (B) vehicle (Veh) and capsaicin (Cap, 3.2 mM). The graphs show the counts for laminae I-V (LI-LV), area X (AX) and the intermediolateral nucleus (IMLN). Means + SEM, n as indicated. * P < 0.05, ** P < 0.01 versus Veh.

In agreement with previous findings [5], IG exposure to 0.5 M HCl failed to induce any expression of c-fos mRNA in the dorsal half of the caudal thoracic spinal cord. Thus, the total number of c-fos mRNA-positive cells per dorsal spinal cord section counted after IG exposure to physiological saline was 1.30 ± 0.29 (n = 4) and after IG exposure to HCl 1.33 ± 0.18 (n = 4). This lack of effect of HCl was also seen when the distribution of c-fos mRNA-positive cells to LI, LII, LIII, LIV, LV, AX and IMLN after IG administration of HCl was compared with that after IG administration of physiological saline (Figure 2A). IG administration of capsaicin (3.2 mM) likewise failed to significantly increase the expression of c-fos mRNA in the dorsal spinal cord, given that the total number of c-fos mRNA-positive cells per section counted after IG exposure to vehicle was 2.19 ± 0.32 (n = 6) and after IG exposure to capsaicin was 1.94 ± 0.23 (n = 7). Analysis of the distribution of c-fos mRNA-positive cells to LI, LII, LIII, LIV, LV, AX and IMLN revealed, however, that capsaicin caused a significant 5.2-fold increase of c-fos mRNA expression in LI, which went in parallel with a significant decrease in the formation of c-fos mRNA in LIII and LIV (Figure 2B). It should be noted that the level of c-fos transcription was very low, as typically less than 0.7 c-fos mRNA-positive cells per lamina were counted in the 0.01 mm sections of the unilateral dorsal spinal cord (Figure 2). For this reason, the experiments involving HCl and capsaicin were strictly run in parallel with those involving the respective control/vehicle solution (Figure 2).

### Effects of HCl and capsaicin on intragastric pressure and gastric fluid recovery (study 2)

**Figure 3:**
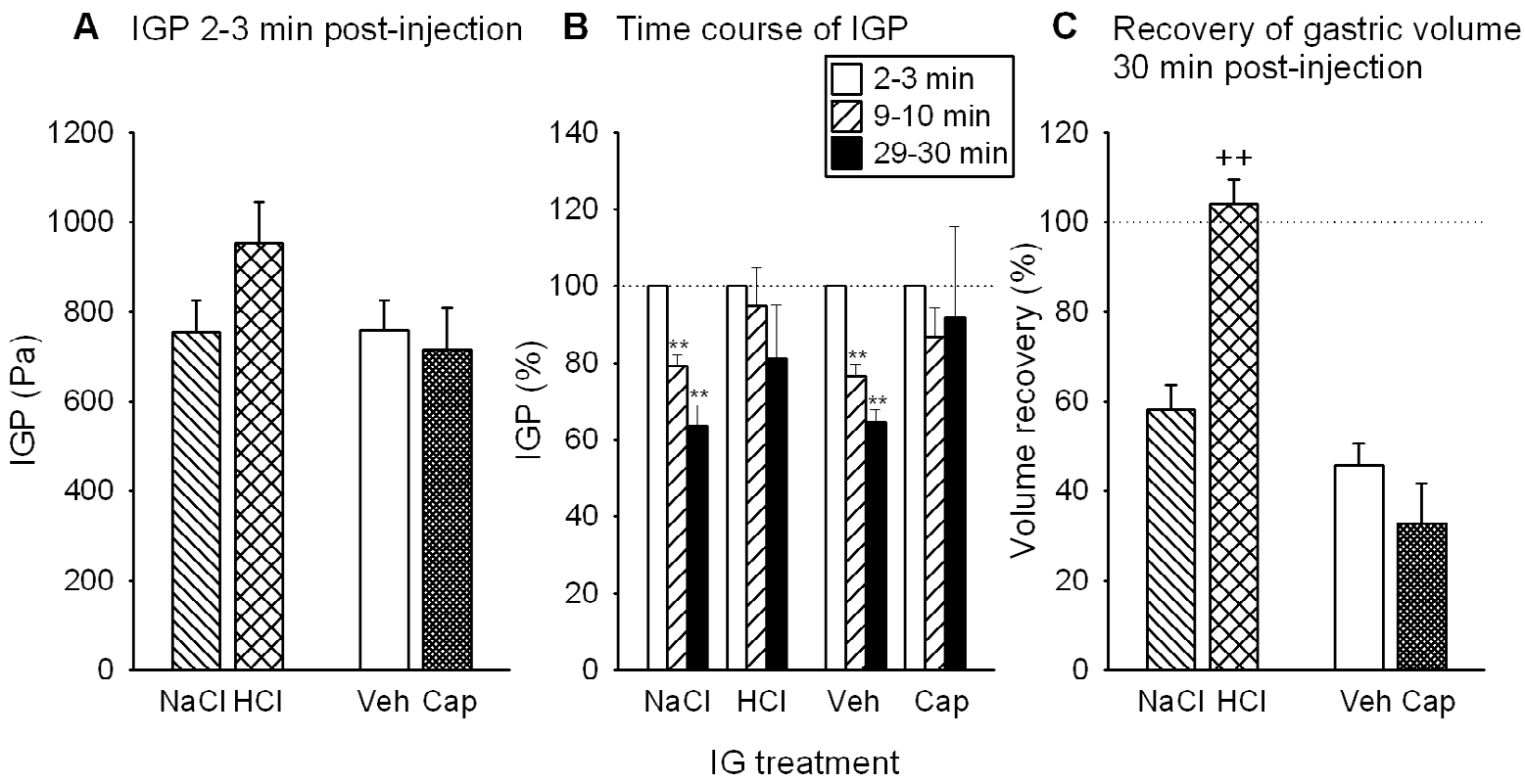
Effects of intragastric injection of NaCl (0.15 M), HCl (0.35 M), vehicle (Veh) and capsaicin (Cap, 3.2 mM) on the (A) initial rise of intragastric pressure (IGP), (B) subsequent time course of IGP and (C) fluid recovery from the stomach. NaCl, HCl, Veh and Cap were injected as 2 ml bolus. The values shown in panel A represent the IGP reached 2–3 min post-injection. The IGP values measured during the periods 9–10 min and 29–30 min post-injection (panel B) are expressed as a percentage of the initial IGP rise recorded 2–3 min post-injection, and the gastric volume recovery (panel C) measured 30 min after bolus injection is expressed as a percentage of the injection volume (2 ml). Means + SEM, n = 6–13. ** P < 0.01 versus IGP measured 2–3 min post-injection; ++ P < 0.01 versus NaCl.

The baseline IGP measured before administration of any medium was between 400 and 500 Pa [20]. IG injection of a 2 ml fluid bolus increased IGP to a level whose magnitude was independent of whether the injected fluid was saline, HCl (0.35 M), vehicle or capsaicin (3.2 mM) as determined 2–3 min post-injection (Figure 3A). In contrast, the time course of the subsequent decline of IGP depended on the nature of the administered medium. Following injection of saline or vehicle, IGP decreased at a significantly faster rate than after injection of HCl or capsaicin, respectively (Figure 3B). Thus, in HCl-and capsaicin-exposed stomachs IGP did not significantly fall during the 30 min observation period post-injection, whereas in NaCl- and vehicle-exposed stomachs IGP significantly decreased to levels of about 65 % of the IGP measured 2–3 min post-injection (Figure 3B). Another effect of HCl was to enhance gastric fluid retention as deduced from a 100 % recovery of the injected fluid volume from the stomach 30 min post-injection (Figure 3C). In contrast, 30 min after administration of saline, vehicle or capsaicin only 30–60% of the injected fluid volume was regained (Figure 3C).

## Discussion

The results of the current study show that IG administration of HCl and capsaicin to rats generates differential inputs to the NTS and thoracic spinal cord, which is associated with differential effects on gastric emptying. As described previously [5,7], gastric signalling to the brainstem and spinal cord was visualized by expression of the inducible gene c-fos at the mRNA level, a method that has been established as a standard tool in functional neuroanatomy to delineate the stimulus-evoked activation of neurons [23,24]. Transcription of the c-fos gene begins within minutes after neuronal excitation [23,24] and in the NTS appears to be maximal 45 min after gastric HCl challenge [5]. Although exposure to HCl (0.35–0.7 M) induces gastric mucosal injury in a concentration-related manner, there is evidence that the afferent signalling of gastric HCl challenge is not directly related to the formation of mucosal injury, because the expression of the c-fos gene in the NTS can be stimulated by IG concentrations of HCl that induce little, if any, epithelial damage [5,7]. It has, therefore, been hypothesized [5,7] that a massive increase of the H^+ ^ion gradient across the gastric mucosal barrier is per se sufficient to drive H^+ ^ions into the lamina propria where they can excite vagal afferent nerve fibres either directly [25,26] or indirectly via neuroactive factors released in the tissue.

The topographical distribution of c-fos mRNA-positive cells in the NTS was uneven but similar after IG administration of HCl and capsaicin. As previously shown by immunohistochemistry [22], the highest number of HCl-activated neurons was seen in the ventromedial part of the NTS. Apart from vagal gastric input [27], this area of the NTS also receives input from spinal lamina I neurons via the spinosolitary tract [28-30]. The relative contribution of the vagal and spinal inputs to this part of the NTS following chemical stimulation of the stomach remains to be determined.

The present study confirms that excess gastric HCl fails to induce c-fos mRNA and c-Fos protein in the dorsal horn of the posterior thoracic spinal cord [5,7] which receives the densest input from afferent neurons innervating the rat stomach [17,18]. Similar findings have been made following exposure of the rat gastric mucosa to ammonium hydroxide [7]. It thus appeared as if gastric challenge with noxious chemicals is signalled by vagal afferents only, a conjecture that was rejected by the current finding that afferent input from the capsaicin-exposed stomach is sent both to the spinal cord and brainstem. This result is in keeping with the expression of TRPV1, the capsaicin receptor, by both vagal and spinal afferent neurons innervating the rat gastrointestinal tract [12-16]. Retrograde tracing has shown that 80 and 71 % of the nodose and dorsal root ganglion neurons supplying the rat stomach, respectively, express TRPV1 [16]. When directly applied to the somata, capsaicin excites 90 % of the dorsal root ganglion neurons and 59 % of the nodose ganglion neurons projecting to the rat stomach [31]. While TRPV1 is easy to detect in the nodose ganglia, the level of TRPV1 expression in most vagal afferent nerve fibres within the stomach is below the immunohistochemical detection threshold [12]. This instance could explain why IG administration of capsaicin induces comparatively little expression of c-fos mRNA in the NTS. We do not think that the small effect of capsaicin is due to inadequate dosing, because it has previously been found that IG administration of 0.64 mM capsaicin is maximally effective in increasing gastric mucosal blood flow in a sensory neuron-dependent manner [19].

Like HCl, capsaicin administered into the rat stomach failed to significantly enhance the overall expression of c-fos mRNA in the dorsal half of the posterior thoracic spinal cord. However, regional analysis revealed that capsaicin caused neurons in the superficial lamina I of the dorsal horn to express c-fos mRNA, an effect that was not seen following IG administration of HCl. This finding obtained with capsaicin is consistent with the projection of visceral afferent neurons to lamina I and the superficial part of lamina II as well as to lamina V and area X of the rat and cat spinal cord [17,32]. Our data indicate that administration of capsaicin into the gastric lumen activates sensory neurons that project primarily to lamina I of the spinal cord. In view of this finding it can be ruled out that c-fos expression is an inadequate method to visualize chemoreceptor signalling from the gastric lumen to the spinal cord and that the failure of gastric HCl challenge to induce c-fos mRNA in the spinal cord represents a false negative result. Although the neural sensors whereby excess HCl is detected in the gastric lumen are not known, it is conceivable that both TRPV1 and acid-sensing ion channels (ASICs) such as ASIC3 are involved [33]. Since both TRPV1 and ASIC3 are expressed by a majority of dorsal root ganglion neurons supplying the rat stomach [16], it appears unlikely that IG HCl fails to induce c-fos mRNA in the spinal cord because the respective afferents do not bear the appropriate acid sensors.

Compared with the number of neurons expressing c-fos mRNA in the NTS, the number of c-fos mRNA-positive neurons in 0.01 mm sections of the spinal cord was very small. This is likely to reflect that the afferent input from the rat stomach to the spinal cord is minor relative to the spinal input from somatic tissues and that electrophysiologically characterized spinal afferents hardly innervate the mucosa of the gastrointestinal tract [34,35]. The low level of c-fos transcription in the laminae of the dorsal spinal cord made it compulsory to run the experiments involving HCl and capsaicin strictly in parallel with those involving the respective control/vehicle solution. We hypothesize that the apparently different distribution of c-fos mRNA-positive cells within the dorsal spinal cord of control rats, as shown in the two panels of Figure 2, may not only reflect inter-experiment variability but also the different nature of the control/vehicle solution: while the vehicle for HCl was physiological saline, the vehicle for capsaicin was saline containing ethanol and Tween 80.

The effect of IG capsaicin to increase c-fos mRNA induction in lamina I of the spinal cord was associated with a significant decrease in c-fos mRNA expression in laminae III and IV. We do not have any straight-forward explanation for this observation. Since laminae III and IV do not seem to receive direct input from the viscera [17,32], we hypothesize that the reduction of c-fos mRNA formation in these laminae is an indirect effect of capsaicin. Conceivably, visceral afferent input via lamina I neurons activates inhibitory pathways that depress the excitability of lamina III and IV neurons.

The afferent signalling of gastric mucosal exposure to capsaicin and excess HCl is determined not only by the concentration of the noxious chemical but also by the duration of its presence in the gastric lumen. It has previously been found that, relative to saline, IG administration of HCl to anaesthetized rats prolongs fluid retention in the stomach and delays adaptation of IGP [7,20]. HCl-induced gastric fluid retention results from inhibition of gastric emptying and enhanced gastric fluid, bicarbonate and mucus secretion [20,36], but analysis of the gastric contents was beyond the scope of this study. HCl-evoked inhibition of gastric emptying is mediated by neural reflexes that are initiated both in the stomach and duodenum [20,37-40]. The concentration of HCl tested for its gastropyloric motor effects in anaesthetized rats was reduced to 0.35 M, because anaesthesia weakens the gastric mucosal barrier to HCl and the IG concentration of HCl (0.5 M) tested for its effect on central c-fos expression induces extensive injury in anaesthetized rats but causes minor gastric damage in conscious animals [5]. As the experiments revealed, gastric exposure to HCl and capsaicin modified gastropyloric motility in a differential manner. While gastric emptying was blocked by HCl but left unaltered by capsaicin, the adaptation of IGP was prevented by both HCl and capsaicin. The effect of capsaicin to delay IGP adaptation may be related to its ability to induce contraction or relaxation of the rat gastric musculature, the type of response depending on the dose of capsaicin and the gastric region and muscle layer under study [19,41-43].

With regard to the disparate effect of IG HCl and capsaicin on spinal c-fos expression it was particularly important to note that, unlike HCl, capsaicin failed to enhance gastric fluid recovery, which means that gastric emptying occurred unimpaired and IG administered capsaicin was quickly transported into the upper small intestine. It could therefore be argued that the capsaicin-evoked c-fos response in the NTS, which was smaller than that to HCl, and spinal cord are due to capsaicin-evoked excitation of both gastric and intestinal afferents, whereas the excitatory effect of HCl is largely confined to gastric afferents. The failure of gastric HCl challenge to induce c-fos expression in the spinal cord cannot be explained by the reported ability of vagal afferents to activate descending pathways and thereby inhibit afferent input to the spinal cord [44,45], because bilateral chronic vagotomy fails to reveal any increase in spinal c-fos mRNA induction due to gastric HCl challenge [5]. There are other ways to explain the differential ability of IG HCl and capsaicin to induce c-fos mRNA in the NTS and spinal cord, but the analysis of these factors was beyond the scope of this study. For instance, there is evidence that capsaicin is little absorbed in the gastric wall [19], which would also explain why IG administered capsaicin is comparatively weak in stimulating gastric vagal afferents projecting to the NTS, whereas capsaicin transported to the upper small intestine may more easily reach and stimulate spinal afferent nerve terminals in the intestinal lamina propria.

Due to its explorative nature, the current study has its limitations. Thus, the differential effect of IG administered capsaicin and HCl on vagal and spinal afferent pathways is likely to depend not only on the gastric emptying rate and the absorption kinetics of HCl and capsaicin but also on the extent of mucosal injury and the magnitude of mucosal blood flow. While the integrity of the gastric mucosa is disturbed only by HCl [5,7,46] but not by capsaicin [11], gastric mucosal blood flow is elevated by both capsaicin [19] and backdiffusing HCl [46].

## Conclusion

Gastric challenge with HCl and capsaicin is differentially signalled to the NTS and spinal cord, which indicates that the two stimuli are processed by disparate nociceptive afferent pathways. Since HCl inhibits gastric emptying, whereas capsaicin does not, it is inferred that the HCl-evoked afferent input to the NTS is transmitted by vagal afferents in the stomach, while the activation of NTS and spinal lamina I neurons by capsaicin is mediated both by vagal afferents in the stomach and by spinal afferents in the upper small intestine. Further experimentation is needed to determine how these findings relate to gastric chemonociception. In agreement with our c-fos data, nociception elicited by excess gastric HCl is mediated by vagal afferent neurons [4], and it awaits to be examined which afferent pathways relay nociception evoked by gastric capsaicin. Mechanonociception evoked by distension of the stomach is brought about by spinal afferents [4], although expression of c-fos is seen both in the spinal cord and, to a larger extent, in the brainstem [47].

## Methods

### Animals

The study was approved by an ethical committee at the Federal Ministry of Education, Science and Culture of the Republic of Austria and conducted according to the Directive of the European Communities Council of 24 November 1986 (86/609/EEC). The experiments were designed in such a way that the number of animals used and their suffering was minimized. Female age-matched Sprague-Dawley rats (Abteilung für Labortierkunde und -genetik, Medical University of Vienna, Himberg, Austria) weighing 180–220 g were used. They were housed in groups of four in plastic transparent cages under standard conditions; lights were on from 6:00 AM until 6:00 PM.

### Experimental protocols

All experiments took place during the light phase between 8:00 and 12:00 AM. Twenty hours before the begin of the experiments the rats were deprived of food to ensure that the stomach was empty by the time of the experiments, while water was available ad libitum throughout this preparatory phase. In addition, the rats were placed in groups of two on a floor grid to prevent coprophagy. Two studies with different experimental protocols were carried out.

Study 1 was conducted with non-anaesthetized animals. Physiological saline (0.15 M NaCl), HCl (0.5 M), capsaicin (0.64 and 3.2 mM) or its vehicle were administered IG at a volume of 10 ml/kg through a soft infant feeding tube (outer diameter 2.2 mm; Portex, Hythe, UK). After 45 min the rats were euthanized by intraperitoneal injection of an overdose of pentobarbital (200 mg/kg; Intervet, Vienna, Austria) and their brainstem and spinal cord removed quickly. Capsaicin (Sigma, Vienna, Austria) was dissolved in a medium containing 10 % Tween 80, 10 % ethanol and 80 % physiological saline to give stock solutions of 2 and 10 mg/ml (6.4 and 32 mM) capsaicin. These stock solutions were then diluted with physiological saline to give test solutions of 0.64 and 3.2 mM capsaicin, respectively. The vehicle for capsaicin consisted of 1 % Tween 80, 1 % ethanol and 98 % physiological saline.

Study 2 was performed with animals that were anaesthetized with phenobarbital (230 mg/kg intraperitoneally; Sigma) and placed on a thermostated table to maintain their rectal temperature at 37 degrees Celsius [20]. The rats were then fitted with a tracheal cannula to facilitate spontaneous breathing. A cannula in the left jugular vein was used for continuous infusion of physiological saline (1.5 ml/h) to avoid dehydration. After a midline laparotomy an IG catheter (outer diameter: 2.2 mm) was inserted in the stomach via the oesophagus, and the stomach flushed [20]. With its tip being positioned in the corpus region, the catheter was used to record intragastric pressure (IGP) via a pressure transducer as well as to inject fluid into and drain it from the stomach [20]. This method of IGP measurement has been described and validated in a previous study [20]. After an equilibration period of 30 min, a 2 ml fluid bolus was slowly injected into the stomach over a period of 5 s and left in the stomach for a period of 30 min after which the stomach was drained and the weight of the recovered fluid determined. The recovery of fluid from the stomach (an indirect measure of gastric emptying) was expressed as a percentage of the weight of the fluid administered into the stomach [20]. Each rat was subjected to 4 injection/recovery trials at intervals of 15 min during which the stomach was left empty. First, two priming trials with saline were carried out, followed either by a test trial with saline and a test trial with HCl (0.35 M) or by a test trial with vehicle and a test trial with capsaicin (3.2 mM). IGP was averaged for the periods of 2–3 min, 9–10 min and 29–30 min post-injection. Since as described before [20] the peak rise of IGP post-injection varied because of differences in injection speed, the IGP averaged during the period of 2–3 min post-injection was taken as 100 % and the IGP recorded during the subsequent observation periods expressed as a percentage of that reference value.

### In situ hybridization autoradiography

The brainstem and spinal cord were quickly removed and frozen on powdered dry ice. Coronal sections (0.01 mm) were cut serially from the brainstem at the rostrocaudal extension of the area postrema and the caudal thoracic spinal cord with a cryostat [5,6,22]. Every sixth section was processed for in situ hybridization with an oligodeoxyribonucleotide probe labelled at the 3' end with [^35^S]deoxyadenosine 5'(α-thio)triphosphate as described previously [6]. The sections were dipped in Ilford K5 photographic emulsion and, after 18–25 days of exposure in sealed boxes at 4 degrees Celsius, the autoradiograms were developed and the sections counterstained with haematoxylin and coverslipped [6]. The specificity of the procedure was proved by the absence of any hybridization signal when control sections were hybridized with a mixture of labelled probe with a 100-fold excess of unlabelled ('cold') probe.

The autoradiograms were examined in a coded manner with a light microscope (Axiophot, Zeiss, Oberkochen, Germany) coupled to a computerized image analysis system (Imaging, St. Catharines, Ontario, Canada). Cells were considered c-fos mRNA-positive when their grain density was at least 10 times higher than the background [6]. In order to enhance the reliability of the quantitative results, 5 brainstem sections and 7–10 spinal cord sections from each animal were evaluated. These sections were selected such that they were 0.05 mm apart from each other so as to avoid that the same cells were counted twice. The c-fos mRNA-positive cells per section were counted unilaterally in the NTS at the level of the area postrema and in the dorsal half of the spinal cord at the caudal thoracic level (T8–T12). These structures were identified according to Molander and Grant [48] and Paxinos and Watson [49]. In the dorsal half of the spinal cord, the distribution of c-fos mRNA-positive cells to laminae I-V (LI-LV), area X (AX) around the central canal and the intermediolateral nucleus (IMLN) was evaluated according to the rat spinal cytoarchitecture described by Molander et al. [50] and Molander and Grant [48]. Particular care was taken to count equivalent sections in the rostro-caudal axis when different treatment groups were compared with each other [5]. All counts per section for a given area in each animal were averaged to give the number of c-fos mRNA-positive cells per section in that particular area. These average values from each animal were then used to calculate the mean number of positive cells per section in the respective areas of each experimental group [5,6].

### Statistics

All data are presented as means ± SEM, n referring to the number of rats in the respective group. After the normal distribution of the experimental data was revealed by the Kolmogorov Smirnov test, significant differences between the experimental groups were evaluated with Student's t test, one-way analysis of variance or, when repeated measurements were taken, one-way analysis of variance for repeated measures followed by Dunn's test (SPSS 11.5, SPSS, Chicago, IL). Probability values of P < 0.05 were regarded as significant.

## List of abbreviations used

ASIC, acid-sensing ion channel; ANOVA, analysis of variance; AX, area X around the central canal of the spinal cord; HCl, hydrochloric acid; IG, intragastric; IGP, intragastric pressure; LI-LV, laminae I-V of the dorsal spinal horn; IMLN, intermediolateral nucleus; mRNA, messenger ribonucleic acid; NTS, nucleus tractus solitarii; Pa, Pascal; TRPV1, transient receptor potential ion channel of vanilloid type 1

## Authors' contributions

PH conceived the study following discussion with EP and RS, coordinated the experiments and drafted the manuscript in close consultation with RS. RS supervised the in situ hybridization experiments, and EP performed the gastric motility study, carried out the statistical analysis of the data and drafted the figures. All authors read and approved the final manuscript.
